# On the Laplacian spectral radii of Halin graphs

**DOI:** 10.1186/s13660-017-1348-5

**Published:** 2017-04-11

**Authors:** Huicai Jia, Jie Xue

**Affiliations:** 1 Department of Mathematics, School of Information, Renmin University of China, Beijing, P.R. China; 2 College of Science, Henan Institute of Engineering, Zhengzhou, Henan P.R. China; 3 Department of Computer Science and Technology, East China Normal University, Shanghai, P.R. China

**Keywords:** 05C50, 05C12, Halin graphs, Laplacian spectral radius

## Abstract

Let *T* be a tree with at least four vertices, none of which has degree 2, embedded in the plane. A Halin graph is a plane graph constructed by connecting the leaves of *T* into a cycle. Thus the cycle *C* forms the outer face of the Halin graph, with the tree inside it. Let *G* be a Halin graph with order *n*. Denote by $\mu(G)$ the Laplacian spectral radius of *G*. This paper determines all the Halin graphs with $\mu(G)\geq n-4$. Moreover, we obtain the graphs with the first three largest Laplacian spectral radius among all the Halin graphs on *n* vertices.

## Introduction

In this paper, we consider simple and undirected connected graphs. Let $G=G(V,E)$ be a simple graph with *n* vertices and *m* edges. Let $N_{G}(v)$ be the set of vertices adjacent to *v* in *G* and $d(v)=|N_{G}(v)|$ be the degree of *v*. As usual, we denote by Δ and *δ* the maximum and minimum degree of *G*, respectively. Denote by $G[S]$ the induced subgraph of *G*. Let $G-v$ be the graph obtained from *G* by deleting the vertex $v\in V(G)$. Similarly, $G-e$ denote the graph obtained from *G* by deleting an edge $e\in G$. Let $G_{1}$ and $G_{2}$ be two vertex disjoint graphs. The graph $G_{1}\cup G_{2}$ is the graph with vertex set $V(G_{1})\cup V(G_{2})$ and edge set $E(G_{1})\cup E(G_{2})$. The join of graphs $G_{1}$ and $G_{2}$ is the graph $G_{1}\vee G_{2}$ obtained from $G_{1}\cup G_{2}$ by joining each vertex of $G_{1}$ with every vertex of $G_{2}$. As usual, we denote by $P_{n}$, $C_{n}$ and $K_{n}$ the path, cycle and complete graph on *n* vertices, respectively.

**Figure 1:**
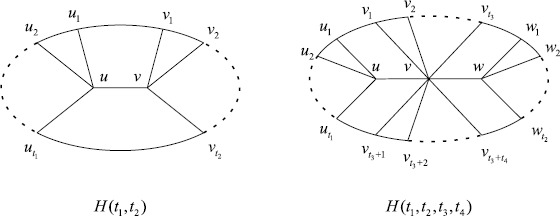
**Halin graphs** $\pmb{H(t_{1},t_{2})}$ **and** $\pmb{H(t_{1},t_{2},t_{3},t_{4})}$ **.**

A *Halin graph* is a plane graph constructed as follows. Let *T* be a tree on at least four vertices. All vertices of *T* have degree 1 or at least 3. The vertices with degree 1 are called *leaves*. Let *C* be a cycle connecting the leaves of *T* in such a way that *C* forms the boundary of the unbounded face. We always say the tree *T* is the characteristic tree of *G* and the cycle *C* is the primary cycle. Moreover, the vertices of *C* are called *exterior* vertices and the other vertices are called *interior* vertices. The Halin graphs was introduced by Halin [1]. We call $K_{1}\vee C_{n-1}$ the *wheel graph*, denoted by $W_{n}$. Clearly, $W_{n}$ is the unique Halin graph with only one interior vertex. In particular, we use $H(t_{1},t_{2})$ and $H(t_{1},t_{2},t_{3},t_{4})$ to denote the Halin graphs with two interior vertices and three interior vertices, respectively (see Figure 1).

For a graph *G*, we assume $d_{1}\geq d_{2}\geq\cdots\geq d_{n}$ is the degree sequence of *G* and $D(G)=\operatorname{diag}(d_{1},d_{2},\ldots,d_{n})$ is the diagonal matrix of vertex degree. Let $A(G)$ be the adjacency matrix. The Laplacian matrix of *G* is defined as $L(G)=D(G)-A(G)$. Obviously, $L(G)$ is a positive semi-definite symmetric matrix, and its eigenvalues are denoted by $\mu_{1}(G)\geq\mu_{2}(G)\geq\cdots\geq\mu _{n}(G)=0$. Moreover, $\mu(G)=\mu_{1}(G)$ is called the *Laplacian spectral radius* of *G*. Let $G^{c}$ be the complement graph of *G*. It is well known that

$$\mu_{i}\bigl(G^{c}\bigr)=n-\mu_{n-i}(G)\quad \text{for }i=1,2,\ldots, n-1. $$

Consequently, we obtain a trivial upper bound of the Laplacian spectral radius: $\mu(G)\leq n$. Let *G* be a Halin graph on *n* vertices, $\mu(G)\geq\Delta(G)+1\geq4$, the equality holds if and only if $G\cong W_{4}$.

The Laplacian eigenvalues of *G* can be used in several physical and chemical problems. Many researchers pay attention to the Laplacian spectra of graphs (see [2–11]). Halin graph is very important in the mathematical literature. In this paper we study the Laplacian spectral radii of Halin graphs. The following are our main results.

### Theorem 1.1

*Let* *G* *be a Halin graph on* *n* *vertices*.

(i) $n\geq\mu(G)> n-1$ *if and only if* $G=W_{n}$.
(ii) $n-1\geq\mu(G)> n-2$ *if and only if* $G=H(n-4,2)$.
(iii) $n-2\geq\mu(G)> n-3$ *if and only if* $G\in\{H(n-5,3),H(2,2,1,0)\}$.
(iv) $n-3\geq\mu(G)\geq n-4$ *if and only if* $G\in\{ H(n-6,4),H(3,2,1,1),H(n-6,2,1,0), H(2,2,t_{3},t_{4})\}$ *where* $t_{3}+t_{4}\geq2$.
(v) *If* $G\notin\{W_{n},H(n-4,2),H(n-5,3)\}$, $\mu(W_{n})>\mu (H(n-4,2))>\mu(H(n-5,3))>\mu(G)$.

## Preliminaries

In order to prove the theorem, we present some lemmas which will be used frequently in the proof.

### Lemma 2.1

[7]

*Let* *G* *be a connected graph on* *n* *vertices with at least one edge*. *Then* $\mu(G)\geq\Delta(G)+1$ *with equality holding if and only if* $\Delta(G)=n-1$.

### Lemma 2.2

[12]

*Let* *G* *be a graph and* $q(G)$ *be the signless Laplacian spectral radius*. *Then* $\mu(G)\leq q(G)$. *Moreover*, *if* *G* *is connected*, *then the equality holds if and only if* *G* *is a bipartite graph*.

### Lemma 2.3

[13]

*Let* *G* *be a simple connected graph with* *n* *vertices and degree sequence* $d_{1}\geq d_{2}\geq\cdots\geq d_{n}$. *Then*

$$q(G)\leq\min_{1\leq i\leq n}\biggl\{ \frac{d_{1}+2d_{i}-1+\sqrt {(2d_{i}-d_{1}+1)^{2}+8(i-1)(d_{1}-d_{i})}}{2}\biggr\} . $$

### Lemma 2.4

[2]

*Let* *G* *be a connected graph*. *Then*

$$\mu(G)\leq\max\bigl\{ d(u)+d(v)\mid uv\in E(G)\bigr\} . $$

*Moreover*, *the equality holds if and only if* *G* *is a regular bipartite graph or a semiregular bipartite graph*.

For a graph *G*, we denote by $m(v)$ the average of degrees of the vertices adjacent to *v*, that is,

$$m(v)=\frac{\sum_{u\in N(v)}d(u)}{d(v)}. $$

As usual, $d(v)m(v)$ is called the 2-*degree* of vertex *v*.

### Lemma 2.5

[6, 8]

*Let* *G* *be a simple graph*. *Then*

$$\mu(G)\leq\max\biggl\{ \frac {d(u)(d(u)+m(u))+d(v)(d(v)+m(v))}{d(u)+d(v)}\biggm|uv\in E(G)\biggr\} . $$

*If* *G* *is connected*, *then equality holds if and only if* *G* *is a regular bipartite graph or a semiregular bipartite graph*.

### Lemma 2.6

[14]

*Let* *G* *be a Halin graph with* *k* *interior vertices*. *Then* $|E(G)|=2n-k-1$ *and* $n\geq2k+2$.

First, we discuss the Halin graphs with at least four interior vertices.

### Lemma 2.7

*Let* *G* *be a Halin graph with* *k* *interior vertices*. *If* $k\geq4$, *then* $\mu(G)< n-4$.

### Proof

Let *G* be a Halin graph with the primary cycle *C*. It follows from Lemma 2.6 that $n\geq2k+2\geq10$. Consider any edge $uv\in E(G)$. If $u,v\in V(C)$, then $d(u)+d(v)=6\leq n-4$. If $u\in V(C)$ and $v \notin V(C)$. Suppose that $d(v)=t+1$. Note that $t+1+3(k-1)-2(k-1)=t+k\geq t+4$, there are at least $t+4$ vertices in *C*. Then $n-k=|V(C)|\geq t+4$, and thus $d(u)+d(v)=t+1+3\leq n-k\leq n-4$. If $u,v\notin V(C)$, and let $d(u)=t_{1}+1$ and $d(v)=t_{2}+1$. Similarly, $t_{1}+1+t_{2}+1+3(k-2)-2(k-1)=t_{1}+t_{2}+k-2\geq t_{1}+t_{2}+2$, so there are at least $t_{1}+t_{2}+2$ vertices in *C*. Then $n-k=|V(C)|\geq t_{1}+t_{2}+2$, and therefore $d(u)+d(v)=t_{1}+1+t_{2}+1\leq n-k\leq n-4$. In each case, we always have $d(u)+d(v)\leq n-4$. It follows from Lemma 2.4 that $\mu(G)< n-4$. □

Next, we consider the Halin graphs with three interior vertices. Let $G=H(t_{1},t_{2},t_{3},t_{4})$ and $t_{1}\geq t_{2}\geq2$. Let *u*, *v* and *w* be three interior vertices. For simplicity, we may take $t=t_{3}+t_{4}\geq1$. It is clear that $d(u)=t_{1}+1$, $d(v)=t+2$, $d(w)=t_{2}+1$ and $n=t+t_{1}+t_{2}+3$.

### Lemma 2.8

*Let* $G=H(t_{1},t_{2},t_{3},t_{4})$ *be a Halin graph with* $t_{1}\geq t_{2}\geq3$. *Then* $\mu(G)< n-4$.

### Proof

For $t_{2}\geq4$, it can easily be seen that $n\geq12$ and

$$\textstyle\begin{cases} t=n-(t_{1}+t_{2}+3)\leq n-11,\\ t_{1}=n-(t+t_{2}+3)\leq n-8,\\ t_{2}=n-(t+t_{1}+3)\leq n-8. \end{cases} $$

Consider all types of edges in *G*. Let $u^{\prime}\in N(u)\cap V(C)$, $v^{\prime}\in N(v)\cap V(C)$ and $w^{\prime}\in N(w)\cap V(C)$. It is obvious that $d(u^{\prime})=d(v^{\prime})=d(w^{\prime})=3$. Then it follows that

$$\begin{aligned} &d(u)+d(v)=t_{1}+t+3=n-t_{2}\leq n-4; \\ &d(v)+d(w)=t_{2}+t+3=n-t_{1}\leq n-4; \\ &d(u)+d\bigl(u^{\prime}\bigr)=t_{1}+1+3=t_{1}+4\leq n-8+4=n-4; \\ &d(w)+d\bigl(w^{\prime}\bigr)=t_{2}+1+3=t_{2}+4\leq n-8+4=n-4; \\ &d(v)+d\bigl(v^{\prime}\bigr)=t+2+3\leq n-11+5=n-6. \end{aligned}$$

If *xy* is an edge in *C*, then $d(x)+d(y)=6\leq n-4$. Consequently, we have $d(x)+d(y)\leq n-4$ for each edge $xy\in E(G)$. Then it follows from Lemma 2.4 that $\mu(G)< n-4$ in this case.

If $t_{2}=3$, then $t_{1}\geq t_{2}=3$ and $n=t+t_{1}+t_{2}+3\geq t_{1}+7$. In this case, we use the bound in Lemma 2.5 to prove the result. Let $u^{\prime}\in N(u)\cap V(C)$, $v^{\prime}\in N(v)\cap V(C)$ and $w^{\prime}\in N(w)\cap V(C)$. Note that

$$\textstyle\begin{cases} d(u)=t_{1}+1,\\ d(v)=t+2=n-t_{1}-4,\\ d(w)=4,\\ d(u^{\prime})=d(v^{\prime})=d(w^{\prime})=3. \end{cases} $$

The 2-degree of each vertex is as follows:

$$\textstyle\begin{cases} d(u)m(u)=n+2t_{1}-4,\\ d(v)m(v)=3n-2t_{1}-13,\\ d(w)m(w)=n-t_{1}+5,\\ d(u^{\prime})m(u^{\prime})=t_{1}+7,\\ d(v^{\prime})m(v^{\prime})=n-t_{1}+2,\\ d(w^{\prime})m(w^{\prime})=10. \end{cases} $$

For all types of edges in *G*, consider the index in Lemma 2.5. Let $e=xy$ be an edge of *G*. Put

$$f(e)=f(xy)=\frac{d(x)(d(x)+m(x))+d(y)(d(y)+m(y))}{d(x)+d(y)}. $$

For simplicity, we use type $u^{\prime}u^{\prime}$ to denote the edges $u_{i}u_{j}\in E(G)$ where $u_{i},u_{j}\in N(u)\cap V(C)$. Similarly, we define the symbol $v^{\prime}v^{\prime}$ and $w^{\prime}w^{\prime}$. Then we will prove that the inequality $f(e)\leq n-4$ holds. Note that each edge of *G* belongs to the one below (the types $u^{\prime }w^{\prime}$ and $v^{\prime}v^{\prime}$ may not exist).

- *uv*:
  $$\begin{aligned} f(uv) =&\frac{d(u)(d(u)+m(u))+d(v)(d(v)+m(v))}{d(u)+d(v)} \\ =&\frac{(t_{1}+1)^{2}+n+2t_{1}-4+(n-t_{1}-4)^{2}+3n-2t_{1}-13}{n-3}. \end{aligned}$$
  Then $f(uv)\leq n-4$ if and only if $(2t_{1}-3)n\geq 2t_{1}^{2}+10t_{1}-12$. Since $n\geq t_{1}+7$, it is easy to verify that $(2t_{1}-3)n\geq(2t_{1}-3)(t_{1}+7)\geq2t_{1}^{2}+10t_{1}-12$ when $t_{1}\geq9$. So we have $f(uv)\leq n-4$ when $t_{1}\geq9$.
  If $t_{1}=8$ and $n\geq16$, then $(2t_{1}-3)n\geq 208>196=2t_{1}^{2}+10t_{1}-12$. Hence $f(uv)\leq n-4$.
  If $t_{1}=7$ and $n\geq15$, then $(2t_{1}-3)n\geq 2t_{1}^{2}+10t_{1}-12$. Hence $f(uv)\leq n-4$.
  An argument similar to the above shows that $f(uv)\leq n-4$ when $\bigl\{ \scriptsize{ \begin{array}{l@{\quad}l} t_{1}=6,\\ n\geq14, \end{array}}$ $\bigl\{ \scriptsize{ \begin{array}{l@{\quad}l} t_{1}=5,\\ n\geq13, \end{array} } $ $\bigl\{ \scriptsize{ \begin{array}{l@{\quad}l} t_{1}=4,\\ n\geq12, \end{array}} $ and $\bigl\{ \scriptsize{ \begin{array}{l@{\quad}l} t_{1}=3,\\ n\geq12. \end{array} } $
  Thus we conclude that inequality $f(uv)\leq n-4$ holds with
  $$\textstyle\begin{cases} t_{1}\geq9,\\ t_{1}=8 \quad\text{and}\quad n\geq16,\\ t_{1}=7 \quad\text{and}\quad n\geq15,\\ t_{1}=6 \quad\text{and}\quad n\geq14,\\ t_{1}=5 \quad\text{and}\quad n\geq13,\\ t_{1}=4 \quad\text{and}\quad n\geq12,\\ t_{1}=3 \quad\text{and}\quad n\geq12. \end{cases} $$
- *vw*:
  $$f(vw)=\frac{(n-t_{1}-4)^{2}+3n-2t_{1}-13+16+n-t_{1}+5}{n-t_{1}}. $$
  Then $f(vw)\leq n-4$ if and only if $t_{1}n\geq t_{1}^{2}+t_{1}+24$. The inequality $f(vw)\leq n-4$ holds with
  $$\textstyle\begin{cases} t_{1}\geq4,\\ t_{1}=3 \quad\text{and}\quad n\geq12. \end{cases} $$
- $uu^{\prime}$:
  $$f\bigl(uu^{\prime}\bigr)=\frac{(t_{1}+1)^{2}+n+2t_{1}-4+9+t_{1}+7}{t_{1}+4}. $$
  Then $f(uu^{\prime})\leq n-4$ if and only if $(t_{1}+3)n\geq t_{1}^{2}+9t_{1}+29$. The inequality $f(uu^{\prime})\leq n-4$ holds with
  $$\textstyle\begin{cases} t_{1}\geq8,\\ t_{1}=7 \quad\text{and}\quad n\geq15,\\ t_{1}=6 \quad\text{and}\quad n\geq14,\\ t_{1}=5 \quad\text{and}\quad n\geq13,\\ t_{1}=4 \quad\text{and}\quad n\geq12,\\ t_{1}=3 \quad\text{and}\quad n\geq11. \end{cases} $$
- $vv^{\prime}$:
  $$f\bigl(vv^{\prime}\bigr)=\frac{(n-t_{1}-4)^{2}+3n-2t_{1}-13+9+n-t_{1}+2}{n-t_{1}-1}. $$
  Then $f(vv^{\prime})\leq n-4$ if and only if $(t_{1}-1)n\geq t_{1}^{2}+t_{1}+10$. The inequality $f(vv^{\prime})\leq n-4$ holds with
  $$\textstyle\begin{cases} t_{1}\geq4,\\ t_{1}=3 \quad\text{and}\quad n\geq11. \end{cases} $$
- $ww^{\prime}$:
  $$f\bigl(ww^{\prime}\bigr)=\frac{n-t_{1}+40}{7}. $$
  Then $f(ww^{\prime})\leq n-4$ if and only if $6n+t_{1}\geq68$. The inequality $f(ww^{\prime})\leq n-4$ holds with
  $$\textstyle\begin{cases} t_{1}\geq4,\\ t_{1}=3 \quad\text{and}\quad n\geq11. \end{cases} $$
- $u^{\prime}v^{\prime}$:
  $$f\bigl(u^{\prime}v^{\prime}\bigr)=\frac{n+27}{6}. $$
  Then $f(u^{\prime}v^{\prime})\leq n-4$ if and only if $5n\geq51$. The inequality $f(u^{\prime}v^{\prime})\leq n-4$ holds with
  $$\textstyle\begin{cases} t_{1}\geq4,\\ t_{1}=3 \quad\text{and}\quad n\geq11. \end{cases} $$
- $v^{\prime}w^{\prime}$:
  $$f\bigl(v^{\prime}w^{\prime}\bigr)=\frac{n-t_{1}+30}{6}. $$
  Then $f(v^{\prime}w^{\prime})\leq n-4$ if and only if $5n+t_{1}\geq 54$. The inequality $f(v^{\prime}w^{\prime})\leq n-4$ holds with
  $$\textstyle\begin{cases} t_{1}\geq4,\\ t_{1}=3 \quad\text{and}\quad n\geq11. \end{cases} $$
- $u^{\prime}w^{\prime}$:
  $$f\bigl(u^{\prime}w^{\prime}\bigr)=\frac{t_{1}+35}{6}. $$
  Then $f(u^{\prime}w^{\prime})\leq n-4$ if and only if $6n-t_{1}\geq 59$. The inequality $f(u^{\prime}w^{\prime})\leq n-4$ holds with
  $$\textstyle\begin{cases} t_{1}\geq4,\\ t_{1}=3 \quad\text{and}\quad n\geq11. \end{cases} $$
- $u^{\prime}u^{\prime}$:
  $$f\bigl(u^{\prime}u^{\prime}\bigr)=\frac{9+t_{1}+7}{3}. $$
  Then $f(u^{\prime}u^{\prime})\leq n-4$ if and only if $3n-t_{1}\geq 28$. The inequality $f(u^{\prime}u^{\prime})\leq n-4$ holds with
  $$\textstyle\begin{cases} t_{1}\geq4,\\ t_{1}=3 \quad\text{and}\quad n\geq11. \end{cases} $$
- $v^{\prime}v^{\prime}$:
  $$f\bigl(v^{\prime}v^{\prime}\bigr)=\frac{9+n-t_{1}+2}{3}. $$
  Then $f(v^{\prime}v^{\prime})\leq n-4$ if and only if $2n+t_{1}\geq 23$. The inequality $f(v^{\prime}v^{\prime})\leq n-4$ holds with
  $$t_{1}\geq3. $$
- $w^{\prime}w^{\prime}$:
  $$f\bigl(w^{\prime}w^{\prime}\bigr)=\frac{19}{3}. $$
  Then $f(w^{\prime}w^{\prime})\leq n-4$ if and only if $3n\geq31$. The inequality $f(v^{\prime}v^{\prime})\leq n-4$ holds with
  $$\textstyle\begin{cases} t_{1}\geq4,\\ t_{1}=3 \quad\text{and}\quad n\geq11. \end{cases} $$

We summarize what has been discussed above as follows.

- If $t_{1}\geq9$, then $\max\{f(e)|e\in E(G)\}\leq n-4$. Moreover, since *G* is not a bipartite graph, it follows from Lemma 2.5 that $\mu(G)< n-4$.
- If $t_{1}=8$, then $n\geq15$. When $n\geq16$, we have $\max\{ f(e)|e\in E(G)\}\leq n-4$. Hence $\mu(G)< n-4$. When $n=15$, that is, $G=H(8,3,1,0)$. Note that $\mu(H(8,3,1,0))\approx10.0680< n-4$. Thus $\mu(G)< n-4$ when $t_{1}=8$.
- If $t_{1}=7$, then $n\geq14$. When $n\geq15$, we infer that $\max\{ f(e)|e\in E(G)\}\leq n-4$. If $n=14$, then $G=H(7,3,1,0)$. Since $\mu (H(7,3,1,0))\approx9.0913< n-4$, it follows that $\mu(G)< n-4$ when $t_{1}=7$.
- If $t_{1}=6$, then $n\geq13$. When $n\geq14$, we have $\max\{ f(e)|e\in E(G)\}\leq n-4$. If $n=13$, then $G=H(6,3,1,0)$. By the fact that $\mu(H(6,3,1,0))\approx8.1298< n-4$, it follows that $\mu(G)< n-4$ when $t_{1}=6$.
- If $t_{1}=5$, then $n\geq12$. When $n\geq13$, we infer that $\max\{ f(e)|e\in E(G)\}\leq n-4$. If $n=12$, then $G=H(5,3,1,0)$. Since $\mu (H(5,3,1,0))\approx7.2022< n-4$, it follows that $\mu(G)< n-4$ when $t_{1}=5$.
- If $t_{1}=4$, then $n\geq11$. When $n\geq12$, we infer that $\max\{ f(e)|e\in E(G)\}\leq n-4$. If $n=11$, then $G=H(4,3,1,0)$. Now that $\mu (H(4,3,1,0))\approx6.3694< n-4$, it follows that $\mu(G)< n-4$ when $t_{1}=4$.
- If $t_{1}=3$, then $n\geq10$. When $n\geq12$, we infer that $\max\{ f(e)|e\in E(G)\}\leq n-4$. If $n=11$, then $G=H(3,3,2,0)$ or $H(3,3,1,1)$. If $n=10$, then $G=H(3,3,1,0)$. Note that $\mu (H(3,3,2,0))\approx6.1116< n-4$, $\mu(H(3,3,1,1))\approx6.4142< n-4$ and $\mu(H(3,3,1,0))\approx5.8577< n-4$. Therefore $\mu(G)< n-4$ in this case.

Thus we have derived that $\mu(G)< n-4$ when $t_{2}=3$. This completes the proof. □

### Lemma 2.9

*Let* $G=H(t_{1},t_{2},t_{3},t_{4})$ *be a Halin graph with* $t_{1}\geq t_{2}=2$.

1. *If* $t_{1}=2$ *or* $n-6$, *then* $\mu(G)>n-4$.
2. *If* $3\leq t_{1}\leq n-7$ *and* $G\neq H(3,2,1,1)$, *then* $\mu(G)< n-4$.

### Proof

For $t_{2}=2$. If $t_{1}=2$ or $n-6$, then $\Delta(G)=n-5$. According to Lemma 2.1, it follows that $\mu(G)>\Delta+1=n-4$. Therefore (1) holds.

Suppose $3\leq t_{1}\leq n-7$. Obviously, $n\geq t_{1}+7$. We also use the bound in Lemma 2.5 to prove the result. Let $u^{\prime}\in N(u)\cap V(C)$, $v^{\prime}\in N(v)\cap V(C)$ and $w^{\prime}\in N(w)\cap V(C)$. Note that

$$\textstyle\begin{cases} d(u)=t_{1}+1,\\ d(v)=n-t_{1}-3,\\ d(w)=3,\\ d(u^{\prime})=d(v^{\prime})=d(w^{\prime})=3. \end{cases} $$

Then the 2-degree of each vertex is as follows:

$$\textstyle\begin{cases} d(u)m(u)=n+2t_{1}-3,\\ d(v)m(v)=3n-2t_{1}-11,\\ d(w)m(w)=n-t_{1}+3,\\ d(u^{\prime})m(u^{\prime})=t_{1}+7,\\ d(v^{\prime})m(v^{\prime})=n-t_{1}+3,\\ d(w^{\prime})m(w^{\prime})=9. \end{cases} $$

For all types of edges in *G*, consider the index in Lemma 2.5. Let $e=xy$ be any one edge of *G*. Put

$$f(e)=f(xy)=\frac{d(x)(d(x)+m(x))+d(y)(d(y)+m(y))}{d(x)+d(y)}. $$

For simplicity, we use $u^{\prime}u^{\prime}$ to denote the edges $u^{\prime}u^{\prime\prime}\in E(G)$ where $u^{\prime},u^{\prime\prime }\in N(u)\cap V(C)$. Similarly, we define the symbol $v^{\prime }v^{\prime}$ and $w^{\prime}w^{\prime}$. Then we will prove that the inequality $f(e)\leq n-4$ holds. Note that every edge of *G* belongs to the one below, and the types $u^{\prime}w^{\prime}$ and $v^{\prime }v^{\prime}$ exist in some circumstances.

- *uv*:
  $$f(uv)=\frac{(t_{1}+1)^{2}+n+2t_{1}-3+(n-t_{1}-3)^{2}+3n-2t_{1}-11}{n-2}. $$
  Then $f(uv)\leq n-4$ if and only if $(t_{1}-2)n\geq t_{1}^{2}+4t_{1}-6$. The inequality $f(uv)\leq n-4$ holds with
  $$\textstyle\begin{cases} t_{1}\geq8,\\ t_{1}=7 \quad\text{and}\quad n\geq15,\\ t_{1}=6 \quad\text{and}\quad n\geq14,\\ t_{1}=5 \quad\text{and}\quad n\geq13,\\ t_{1}=4 \quad\text{and}\quad n\geq13,\\ t_{1}=3 \quad\text{and}\quad n\geq15. \end{cases} $$
- *vw*:
  $$f(vw)=\frac{(n-t_{1}-3)^{2}+3n-2t_{1}-11+9+n-t_{1}+3}{n-t_{1}}. $$
  Then $f(vw)\leq n-4$ if and only if $(t_{1}-2)n\geq t_{1}^{2}-t_{1}+10$. The inequality $f(vw)\leq n-4$ holds with
  $$\textstyle\begin{cases} t_{1}\geq4,\\ t_{1}=3 \quad\text{and}\quad n\geq16. \end{cases} $$
- $uu^{\prime}$:
  $$f\bigl(uu^{\prime}\bigr)=\frac{(t_{1}+1)^{2}+n+2t_{1}-3+9+t_{1}+7}{t_{1}+4}. $$
  Then $f(uu^{\prime})\leq n-4$ if and only if $(t_{1}+3)n\geq t_{1}^{2}+9t_{1}+30$. The inequality $f(uu^{\prime})\leq n-4$ holds with
  $$\textstyle\begin{cases} t_{1}\geq9,\\ t_{1}=8 \quad\text{and}\quad n\geq16,\\ t_{1}=7 \quad\text{and}\quad n\geq15,\\ t_{1}=6 \quad\text{and}\quad n\geq14,\\ t_{1}=5 \quad\text{and}\quad n\geq13,\\ t_{1}=4 \quad\text{and}\quad n\geq12,\\ t_{1}=3 \quad\text{and}\quad n\geq11. \end{cases} $$
- $vv^{\prime}$:
  $$f\bigl(vv^{\prime}\bigr)=\frac{(n-t_{1}-3)^{2}+3n-2t_{1}-11+9+n-t_{1}+3}{n-t_{1}}. $$
  Then $f(vv^{\prime})\leq n-4$ if and only if $(t_{1}-2)n\geq t_{1}^{2}-t_{1}+10$. The inequality $f(vv^{\prime})\leq n-4$ holds with
  $$\textstyle\begin{cases} t_{1}\geq4,\\ t_{1}=3 \quad\text{and}\quad n\geq16. \end{cases} $$
- $ww^{\prime}$:
  $$f\bigl(ww^{\prime}\bigr)=\frac{n-t_{1}+30}{6}. $$
  Then $f(ww^{\prime})\leq n-4$ if and only if $5n+t_{1}\geq54$. The inequality $f(ww^{\prime})\leq n-4$ holds with
  $$\textstyle\begin{cases} t_{1}\geq4,\\ t_{1}=3 \quad\text{and}\quad n\geq11. \end{cases} $$
- $u^{\prime}v^{\prime}$:
  $$f\bigl(u^{\prime}v^{\prime}\bigr)=\frac{n+28}{6}. $$
  Then $f(u^{\prime}v^{\prime})\leq n-4$ if and only if $5n\geq52$. The inequality $f(u^{\prime}v^{\prime})\leq n-4$ holds with
  $$\textstyle\begin{cases} t_{1}\geq4,\\ t_{1}=3 \quad\text{and}\quad n\geq11. \end{cases} $$
- $v^{\prime}w^{\prime}$:
  $$f\bigl(v^{\prime}w^{\prime}\bigr)=\frac{n-t_{1}+30}{6}. $$
  Then $f(v^{\prime}w^{\prime})\leq n-4$ if and only if $5n+t_{1}\geq 54$. The inequality $f(v^{\prime}w^{\prime})\leq n-4$ holds with
  $$\textstyle\begin{cases} t_{1}\geq4,\\ t_{1}=3 \quad\text{and}\quad n\geq11. \end{cases} $$
- $u^{\prime}w^{\prime}$:
  $$f\bigl(u^{\prime}w^{\prime}\bigr)=\frac{t_{1}+34}{6}. $$
  Then $f(u^{\prime}w^{\prime})\leq n-4$ if and only if $6n-t_{1}\geq 58$. The inequality $f(u^{\prime}w^{\prime})\leq n-4$ holds with
  $$\textstyle\begin{cases} t_{1}\geq4,\\ t_{1}=3 \quad\text{and}\quad n\geq11. \end{cases} $$
- $u^{\prime}u^{\prime}$:
  $$f\bigl(u^{\prime}u^{\prime}\bigr)=\frac{9+t_{1}+7}{3}. $$
  Then $f(u^{\prime}u^{\prime})\leq n-4$ if and only if $3n-t_{1}\geq 28$. The inequality $f(u^{\prime}u^{\prime})\leq n-4$ holds with
  $$\textstyle\begin{cases} t_{1}\geq4,\\ t_{1}=3 \quad\text{and}\quad n\geq11. \end{cases} $$
- $v^{\prime}v^{\prime}$:
  $$f\bigl(v^{\prime}v^{\prime}\bigr)=\frac{9+n-t_{1}+3}{3}. $$
  Then $f(v^{\prime}v^{\prime})\leq n-4$ if and only if $2n+t_{1}\geq 24$. The inequality $f(v^{\prime}v^{\prime})\leq n-4$ holds with
  $$\textstyle\begin{cases} t_{1}\geq4,\\ t_{1}=3 \quad\text{and}\quad n\geq11. \end{cases} $$
- $w^{\prime}w^{\prime}$:
  $$f\bigl(w^{\prime}w^{\prime}\bigr)=6. $$
  Since $n\geq t_{1}+7\geq10$, we have $f(w^{\prime}w^{\prime})\leq n-4$.

So we have the following conclusions.

- If $t_{1}\geq9$, then $\max\{f(e)|e\in E(G)\}\leq n-4$. According to Lemma 2.5, it follows that $\mu(G)< n-4$.
- If $t_{1}=8$, then $n\geq15$. When $n\geq16$, we have $\max\{ f(e)|e\in E(G)\}\leq n-4$. If $n=15$, then $G=H(8,2,2,0)$ or $H(8,2,1,1)$. Note that $\mu(H(8,2,2,0))\approx10.0928< n-4$ and $\mu (H(8,2,1,1))\approx10.1016< n-4$. Thus $\mu(G)< n-4$ when $t_{1}=8$.
- If $t_{1}=7$, then $n\geq14$. When $n\geq15$, we have $\max\{ f(e)|e\in E(G)\}\leq n-4$. If $n=14$, then $G=H(7,2,2,0)$ or $H(7,2,1,1)$. Note that $\mu(H(7,2,2,0))\approx9.1261< n-4$ and $\mu (H(7,2,1,1))\approx9.1414< n-4$. Hence $\mu(G)< n-4$ when $t_{1}=7$.
- If $t_{1}=6$, then $n\geq13$. When $n\geq14$, we have $\max\{ f(e)|e\in E(G)\}\leq n-4$. If $n=13$, then $G=H(6,2,2,0)$ or $H(6,2,1,1)$. Note that $\mu(H(6,2,2,0))\approx8.1820< n-4$ and $\mu (H(6,2,1,1))\approx8.2113< n-4$. Hence $\mu(G)< n-4$ when $t_{1}=6$.
- If $t_{1}=5$, then $n\geq12$. When $n\geq13$, we have $\max\{ f(e)|e\in E(G)\}\leq n-4$. If $n=12$, then $G=H(5,2,2,0)$ or $H(5,2,1,1)$. Note that $\mu(H(5,2,2,0))\approx7.2861< n-4$ and $\mu (H(5,2,1,1))\approx7.3502< n-4$. Hence $\mu(G)< n-4$ when $t_{1}=5$.
- If $t_{1}=4$, then $n\geq11$. When $n\geq13$, we have $\max\{ f(e)|e\in E(G)\}\leq n-4$. If $n=12$, then $G=H(4,2,3,0)$ or $H(4,2,2,1)$. If $n=11$, then $G=H(4,2,2,0)$ or $H(4,2,1,1)$. Note that $\mu(H(4,2,3,0))\approx6.8985< n-4$, $\mu(H(4,2,2,1))\approx 7.0131< n-4$, $\mu(H(4,2,2,0))\approx6.5037< n-4$ and $\mu (H(4,2,1,1))\approx6.6518< n-4$. Therefore $\mu(G)< n-4$ when $t_{1}=4$.
- If $t_{1}=3$, then $n\geq10$. When $n\geq15$, we have $\max\{ f(e)|e\in E(G)\}\leq n-4$. If $n=10, 11, 12, 13, 14$, then $G\in\mathbb{H}=\{ H(3,2,2,0),H(3,2,1,1),H(3,2,3,0),H(3,2,2,1), H(3,2,4,0), H(3,2,3,1),H(3,2,2,2),H(3,2,5,0),H(3,2,4,1),H(3,2,3,2), H(3,2,6,0), H(3,2,5,1),H(3,2,4,2),H(3,2,3,3)\}$.
  Note that $\mu(H(3,2,1,1))\approx6.2470>n-4$ and if $G\in\mathbb {H}\backslash\{H(3,2,1,1)\}$ then $\mu(G)< n-4$ (see Table 1). This implies that if $t_{1}=3$ and $G\neq H(3,2,1,1)$, then $\mu(G)< n-4$.

**Table 1:** **The Laplacian spectral radii of some Halin graphs with three interior vertices**

	***G***	***μ*** **(** ***G*** **)**
*n* = 8	*H*(2,2,1,0)	5.4142
*n* = 9	*H*(2,2,2,0)	5.6996
	*H*(2,2,1,1)	6.0000
	*H*(3,2,1,0)	5.7480
*n* = 10	*H*(3,3,1,0)	5.8577
	*H*(2,2,3,0)	6.4423
	*H*(2,2,2,1)	6.5846
	*H*(4,2,1,0)	6.3500
	*H*(3,2,2,0)	5.9709
	*H*(3,2,1,1)	6.2470
*n* = 11	*H*(3,3,2,0)	6.1116
	*H*(3,3,1,1)	6.4142
	*H*(4,3,1,0)	6.3694
	*H*(3,2,3,0)	6.5894
	*H*(3,2,2,1)	6.7387
	*H*(4,2,2,0)	6.5037
	*H*(4,2,1,1)	6.6518
*n* = 12	*H*(5,3,1,0)	7.2022
	*H*(3,2,4,0)	7.3612
	*H*(3,2,3,1)	7.4771
	*H*(3,2,2,2)	7.4446
	*H*(4,2,3,0)	6.8985
	*H*(4,2,2,1)	7.0131
	*H*(5,2,2,0)	7.2861
	*H*(5,2,1,1)	7.3502
*n* = 13	*H*(6,3,1,0)	8.1298
	*H*(3,2,5,0)	8.2480
	*H*(3,2,4,1)	8.3198
	*H*(3,2,3,2)	8.3052
	*H*(6,2,2,0)	8.1820
	*H*(6,2,1,1)	8.2113
*n* = 14	*H*(7,3,1,0)	9.0913
	*H*(3,2,6,0)	9.1708
	*H*(3,2,5,1)	9.2272
	*H*(3,2,4,2)	9.2180
	*H*(3,2,3,3)	9.2198
	*H*(7,2,2,0)	9.1261
	*H*(7,2,1,1)	9.1414
*n* = 15	*H*(8,3,1,0)	10.0680
	*H*(8,2,2,0)	10.0928
	*H*(8,2,1,1)	10.1016

Consequently, we infer that (2) holds. This completes the proof. □

For Halin graphs with three interior vertices. From the proof of the above lemmas, we see that only $H(3,2,1,1)$, $H(2,2,t_{3},t_{4})$ and $H(n-6,2,1,0)$ have the Laplacian spectral radii greater than $n-4$. Clearly, $n-4<\mu(H(3,2,1,1))<n-3$ (see Table 1).

### Lemma 2.10

*Let* $G\in\{H(2,2,t_{3},t_{4}),H(n-6,2,1,0)\}$, *where* $t_{3}+t_{4}\geq 2$, *then* $\mu(G)\leq n-3$. *If* $G=H(2,2,1,0)$, *then* $n-3< \mu(G)<n-2$.

### Proof

It is clear that $H(2,2,t_{3},t_{4})$ and $H(n-6,2,1,0)$ have the same degree sequence:

$$(d_{1},d_{2},\ldots,d_{n})=(n-5,3,3,\ldots, 3). $$

Let $G\in\{H(2,2,t_{3},t_{4}),H(n-6,2,1,0)\}$. By Lemmas 2.2 and 2.3, we have

$$\begin{aligned} \mu(G) < &\min_{1\leq i\leq n}\biggl\{ \frac{d_{1}+2d_{i}-1+\sqrt {(2d_{i}-d_{1}+1)^{2}+8(i-1)(d_{1}-d_{i})}}{2}\biggr\} \\ \leq& \frac{n-5+6-1+\sqrt{(6-(n-5)+1)^{2}+8(n-5-3)}}{2} \\ =&\frac{n+\sqrt{(n-12)^{2}+8(n-8)}}{2}. \end{aligned}$$

If $n\geq11$, it is easy to check that

$$n-3\geq\frac{n+\sqrt{(n-12)^{2}+8(n-8)}}{2}. $$

Therefore $\mu(G)< n-3$. If $8\leq n\leq10$, then $G\in\{H(2,2,1,0), H(2,2,2,0), H(2,2,1,1), H(3,2,1,0), H(2,2,3,0), H(2,2,2,1), H(4,2,1,0)\}$. If $G=H(2,2,1,0)$, then $n-3< \mu(G)<n-2$. Otherwise, $\mu(G)\leq n-3$ (see Table 1). This lemma follows. □

Now we consider the Halin graphs with two interior vertices. Let $G=H(t_{1},t_{2})$ and $t_{1}\geq t_{2}\geq2$. Note that $t_{1}=n-t_{2}-2\geq t_{2}$, then $n\geq2t_{2}+2$.

### Lemma 2.11

*Let* $G=H(t_{1},t_{2})$ *be a Halin graph with* $t_{1}\geq t_{2}\geq5$. *Then* $\mu(G)< n-4$.

### Proof

Suppose *u* and *v* are the two interior vertices. Let $u^{\prime}\in N(u)\cap V(C)$ and $v^{\prime}\in N(v)\cap V(C)$. Note that

$$\textstyle\begin{cases} d(u)=n-t_{2}-1,\\ d(v)=t_{2}+1,\\ d(u^{\prime})=d(v^{\prime})=3. \end{cases} $$

Then the 2-degree of each vertex is as follows:

$$\textstyle\begin{cases} d(u)m(u)=3n-2t_{2}-5,\\ d(v)m(v)=n+2t_{2}-1,\\ d(u^{\prime})m(u^{\prime})=n-t_{2}+5,\\ d(v^{\prime})m(v^{\prime})=t_{2}+7. \end{cases} $$

For all types of edges in *G*, consider the index in Lemma 2.5. Let $e=xy$ be any one edge of *G*. We may take

$$f(e)=f(xy)=\frac{d(x)(d(x)+m(x))+d(y)(d(y)+m(y))}{d(x)+d(y)}. $$

For simplicity, we use $u^{\prime}u^{\prime}$ to denote the edges $u^{\prime}u^{\prime\prime}\in E(G)$ where $u^{\prime},u^{\prime\prime }\in N(u)\cap V(C)$. Similarly, we define the symbol $v^{\prime }v^{\prime}$. It is clear that every edge of *G* belongs to the one below.

- *uv*:
  $$f(uv)=\frac{(n-t_{2}-1)^{2}+3n-2t_{2}-5+(t_{2}+1)^{2}+n+2t_{2}-1}{n}. $$
  Then $f(uv)\leq n-4$ if and only if $(t_{2}-3)n\geq t_{2}^{2}+2t_{2}-2$. The inequality $f(uu^{\prime})\leq n-4$ holds with
  $$\textstyle\begin{cases} t_{2}\geq7,\\ t_{2}=6 \quad\text{and}\quad n\geq16,\\ t_{2}=5 \quad\text{and}\quad n\geq17. \end{cases} $$
- $uu^{\prime}$:
  $$f\bigl(uu^{\prime}\bigr)=\frac{(n-t_{2}-1)^{2}+3n-2t_{2}-5+9+n-t_{2}+5}{n-t_{2}+2}. $$
  Then $f(uu^{\prime})\leq n-4$ if and only if $(t_{2}-4)n\geq t_{2}^{2}-5t_{2}+18$. The inequality $f(uu^{\prime})\leq n-4$ holds with
  $$\textstyle\begin{cases} t_{2}\geq6,\\ t_{2}=5 \quad\text{and}\quad n\geq18. \end{cases} $$
- $vv^{\prime}$:
  $$f\bigl(vv^{\prime}\bigr)=\frac{(t_{2}+1)^{2}+n+2t_{2}-1+9+t_{2}+7}{t_{2}+4}. $$
  Then $f(vv^{\prime})\leq n-4$ if and only if $(t_{2}+3)n\geq t_{2}^{2}+9t_{2}+32$. The inequality $f(vv^{\prime})\leq n-4$ holds with
  $$\textstyle\begin{cases} t_{2}\geq6,\\ t_{2}=5 \quad\text{and}\quad n\geq13. \end{cases} $$
- $u^{\prime}v^{\prime}$:
  $$f\bigl(u^{\prime}v^{\prime}\bigr)=\frac{n+30}{6}. $$
  Then $f(u^{\prime}v^{\prime})\leq n-4$ if and only if $5n\geq54$. If $t_{2}\geq5$, $f(u^{\prime}v^{\prime})\leq n-4$.
- $u^{\prime}u^{\prime}$:
  $$f\bigl(u^{\prime}u^{\prime}\bigr)=\frac{18+2(n-t_{2}+5)}{6}. $$
  Then $f(u^{\prime}u^{\prime})\leq n-4$ if and only if $2n+t_{2}\geq 26$. If $t_{2}\geq5$, $f(u^{\prime}u^{\prime})\leq n-4$.
- $v^{\prime}v^{\prime}$:
  $$f\bigl(v^{\prime}v^{\prime}\bigr)=\frac{18+2(t_{2}+7)}{6}. $$
  Then $f(v^{\prime}v^{\prime})\leq n-4$ if and only if $3n-t_{2}\geq 28$. If $t_{2}\geq5$, $f(v^{\prime}v^{\prime})\leq n-4$.

Thus we infer that $\max\{f(e)|e\in E(G)\}\leq n-4$ if $t_{2}\geq7$, $\bigl\{ \scriptsize{ \begin{array}{l@{\quad}l} t_{2}=6,\\ n\geq16, \end{array}} $ or $\bigl\{ \scriptsize{ \begin{array}{l@{\quad}l} t_{2}=5,\\ n\geq18. \end{array}} $ According to Lemma 2.5, it follows that $\mu(G)< n-4$. Otherwise,

$$G\in\bigl\{ H(5,5),H(6,5),H(6,6),H(7,5),H(7,6),H(8,5),H(9,5),H(10,5)\bigr\} . $$

It is easy to see that $\mu(G)< n-4$ in this case (see Table 2). This completes the proof.

**Table 2:** **The Laplacian spectral radii of some Halin graphs with two interior vertices**

	***G***	***μ*** **(** ***G*** **)**
*n* = 6	*H*(2,2)	5.0000
*n* = 7	*H*(3,2)	5.6180
*n* = 8	*H*(3,3)	6.0000
	*H*(4,2)	6.3234
*n* = 9	*H*(4,3)	6.5315
*n* = 10	*H*(4,4)	6.8820
	*H*(5,3)	7.3058
*n* = 11	*H*(5,4)	7.4911
	*H*(6,3)	8.1938
*n* = 12	*H*(5,5)	7.8605
	*H*(6,4)	8.2932
	*H*(7,3)	9.1335
*n* = 13	*H*(6,5)	8.4827
	*H*(7,4)	9.1923
	*H*(8,3)	10.0976
*n* = 14	*H*(6,6)	8.8562
	*H*(7,5)	9.2930
	*H*(8,4)	10.1358
*n* = 15	*H*(7,6)	9.4835
	*H*(8,5)	10.1954
	*H*(9,4)	11.1012
*n* = 16	*H*(9,5)	11.1399
	*H*(10,4)	12.0785
*n* = 17	*H*(10,5)	12.1055
	*H*(11,4)	13.0627
*n* = 18	*H*(12,4)	14.0513

□

### Lemma 2.12

*Let* $G=H(n-6,4)$ *be a Halin graph with* $n\geq10$ *vertices*. *Then* $n-4<\mu(G)<n-3$.

### Proof

Since $\Delta(G)=n-5$, it follows from Lemma 2.1 that $\mu (G)>n-4$. The degree sequence of *G* is $(d_{1},d_{2},\ldots ,d_{n})=(n-5,5,3,\ldots, 3)$. From Lemmas 2.2 and 2.3, we have

$$\begin{aligned} \mu(G) < &\min_{1\leq i\leq n}\biggl\{ \frac{d_{1}+2d_{i}-1+\sqrt {(2d_{i}-d_{1}+1)^{2}+8(i-1)(d_{1}-d_{i})}}{2}\biggr\} \\ \leq& \frac{d_{1}+2d_{2}-1+\sqrt {(2d_{2}-d_{1}+1)^{2}+8(d_{1}-d_{2})}}{2} \\ =&\frac{n+4+\sqrt{(n-16)^{2}+8(n-10)}}{2}. \end{aligned}$$

If $n\geq19$, then we get

$$n-3\geq\frac{n+4+\sqrt{(n-16)^{2}+8(n-10)}}{2}. $$

Therefore $\mu(G)< n-3$ when $n\geq19$. If $n\leq18$, then $G=H(t_{1},4)$ where $t_{1}=4,5,6,\ldots,12$. It is easy to check that $\mu(G)< n-3$ (see Table 2). Thus we complete the proof. □

### Lemma 2.13

*Let* $G=H(n-5,3)$ *be a Halin graph with* $n\geq8$ *vertices*. *Then* $n-3<\mu (G)\leq n-2$. *Moreover*, *the right equality holds if and only* $G=H(3,3)$.

### Proof

The degree sequence of *G* is $(d_{1},d_{2},\ldots ,d_{n})=(n-4,4,3,\ldots, 3)$. It follows from Lemma 2.1 that $\mu (G)>\Delta(G)+1=n-3$. From Lemmas 2.2 and 2.3, we have

$$\begin{aligned} \mu(G) < &\min_{1\leq i\leq n}\biggl\{ \frac{d_{1}+2d_{i}-1+\sqrt {(2d_{i}-d_{1}+1)^{2}+8(i-1)(d_{1}-d_{i})}}{2}\biggr\} \\ \leq& \frac{d_{1}+2d_{2}-1+\sqrt {(2d_{2}-d_{1}+1)^{2}+8(d_{1}-d_{2})}}{2} \\ =&\frac{n+3+\sqrt{(n-13)^{2}+8(n-8)}}{2}. \end{aligned}$$

If $n\geq14$, then

$$n-2\geq\frac{n+3+\sqrt{(n-13)^{2}+8(n-8)}}{2}. $$

Therefore $\mu(G)< n-3$. If $n\leq13$, then $G=H(t_{1},3)$ where $t_{1}=3,4,5,\ldots,8$. It is clear that $\mu(H(3,3))=n-2$ and $\mu (H(t_{1},3))< n-2$ where $t_{1}=4,5,\ldots,8$ (see Table 2). Thus we complete the proof. □

### Lemma 2.14

*Let* $G=H(n-4,2)$ *be a Halin graph with* $n\geq6$ *vertices*. *Then* $n-2<\mu (G)\leq n-1$. *Moreover*, *the right equality holds if and only* $G=H(2,2)$.

### Proof

The degree sequence of *G* is $(d_{1},d_{2},\ldots ,d_{n})=(n-3,3,3,\ldots, 3)$. It follows from Lemma 2.1 that $\mu (G)>\Delta(G)+1=n-2$. From Lemmas 2.2 and 2.3, we have

$$\begin{aligned} \mu(G) < &\min_{1\leq i\leq n}\biggl\{ \frac{d_{1}+2d_{i}-1+\sqrt {(2d_{i}-d_{1}+1)^{2}+8(i-1)(d_{1}-d_{i})}}{2}\biggr\} \\ \leq& \frac{d_{1}+2d_{2}-1+\sqrt {(2d_{2}-d_{1}+1)^{2}+8(d_{1}-d_{2})}}{2} \\ =&\frac{n+2+\sqrt{(n-10)^{2}+8(n-6)}}{2}. \end{aligned}$$

If $n\geq9$, then

$$n-1\geq\frac{n+2+\sqrt{(n-10)^{2}+8(n-6)}}{2}. $$

Therefore $\mu(G)< n-1$. If $n\leq8$, then $G=H(2,2)$, $H(3,2)$ or $H(4,2)$. It is clear that $\mu(H(2,2))=n-1$, $\mu(H(3,2))< n-1$ and $\mu (H(4,2))< n-1$ (see Table 2). Thus we complete the proof. □

Now we are ready to present the proof of Theorem 1.1. In fact, from the previous lemmas, it is easy to obtain the main result. For the sake of completeness, we provide a brief proof.

## Proof of Theorem 1.1

Let *G* be a Halin graph. We make a summary of Lemmas 2.6-2.14.

If *G* has $k\geq4$ interior vertices, then $\mu(G)< n-4$.

If *G* has three interior vertices, then $\mu(G)< n-4$ when $G\notin\{ H(2,2,t_{3},t_{4}),H(n-6,2,1,0),H(3,2,1,1)\}$, where $t_{3}+t_{4}\geq1$; if $G\in\{H(2,2,t_{3},t_{4}),H(n-6,2,1,0),H(3,2,1,1)\}$, where $t_{3}+t_{4}\geq2$, then $n-4<\mu(G)\leq n-3$; if $G=H(2,2,1,0)$, then $n-3<\mu(G)<n-2$.

If *G* has two interior vertices, then $\mu(G)< n-4$ when

$$G\notin\bigl\{ H(n-6,4),H(n-5,3),H(n-4,2)\bigr\} . $$

On the other hand, we have $n-4<\mu(H(n-6,4))<n-3$, $n-3<\mu (H(n-5,3))\leq n-2$, $n-2<\mu(H(n-4,2))\leq n-1$ and $\mu(H(n-5,3))>\mu (H(2,2,1,0))$.

If *G* has one interior vertex, then $G=W_{n}$ and $\mu(W_{n})=n$.

It is now obvious that the theorem holds.

### Remark 3.1

From the proof, we see that there is no graph with $n-1<\mu(G)<n$. If $\mu(G)=n-1$ iff $G=H(2,2)$. If $\mu(G)=n-2$ iff $G=H(3,3)$. There is no graph with $\mu(G)=n-4$.

### Remark 3.2

Let $H(n-t-2,t)$ be a Halin with *n* vertices and $n\geq 2t+2$. Then $\Delta=n-t-1$, so $\mu(H(n-t-2,t))>n-t$. The degree sequence is $(n-t-1,t+1,3,\ldots,3)$, then if $n\geq5t-1$, we have

$$\mu\bigl(H(n-t-2,t)\bigr)\leq\frac{d_{1}+2d_{2}-1+\sqrt {(2d_{2}-d_{1}+1)^{2}+8(d_{1}-d_{2})}}{2}\leq n-t+1. $$

That is, for an integer *k*, when *n* is sufficiently large, then $n-t<\mu(H(n-t-2,t))\leq n-t+1$. From this we propose the following conjecture.

### Conjecture 3.1

*Let* $H(t_{1},t_{2})$ *be a Halin graph with two interior vertices and order* *n*, *where* $n=t_{1}+t_{2}+2$ *and* $t_{1}\geq t_{2}$. *Then*

1. $n-t_{2}<\mu(H(t_{1},t_{2}))\leq n-t_{2}+1$;
2. $\mu(H(t_{1},t_{2}))<\mu(H(t_{1}+1,t_{2}-1))$.

## Conclusions

We determine all the Halin graphs with $\mu(G)\geq n-4$. Moreover, we also obtain the graphs with the first three largest Laplacian spectral radius among all the Halin graphs on *n* vertices. Considering the further order of the Laplacian spectral radius of Halin graphs is still an interesting and important problem.

## References

[1] Halin R (1964). Über simpliziable Zerfallungen beliebiger. Math. Ann..

[2] Anderson W, Morley T (1985). Eigenvalues of the Laplacian of a graph. Linear Multilinear Algebra.

[3] Bıyıkoğlu T, Leydold J (2010). Semiregular trees with minimal Laplacian spectral radius. Linear Algebra Appl..

[4] Das KCh (2004). Sharp lower bounds on the Laplacian eigenvalues of trees. Linear Algebra Appl..

[5] Liu M, Liu B (2010). Some results on the Laplacian spectrum. Comput. Math. Appl..

[6] Li J, Zhang X (1998). On Laplacian eigenvalues of a graph. Linear Algebra Appl..

[7] Merris R (1994). Laplacian matrices of graphs: a survey. Linear Algebra Appl..

[8] Pan Y (2002). Sharp upper bounds for the Laplacian graph eigenvalues. Linear Algebra Appl..

[9] Simic SK, Stanic Z (2009). On some forests determined by their Laplacian or signless Laplacian spectrum. Comput. Math. Appl..

[10] Teranishi Y (2011). Subgraphs and the Laplacian spectrum of a graph. Linear Algebra Appl..

[11] Yuan X, Shan H, Liu Y (2009). On the Laplacian spectral radii of trees. Discrete Math..

[12] Zhang X, Luo R (2002). The spectral radius of triangle-free graphs. Australas. J. Comb..

[13] Yu G, Wu Y, Shu J (2011). Sharp bounds on the signless Laplacian spectral radii of graphs. Linear Algebra Appl..

[14] Shu J, Hong Y (2000). The upper bound for the spectral radius of outplanar graphs and Halin graphs. Chin. Ann. Math., Ser. A.

